# Home care-based education for cancer patients with peripherally inserted central catheters: a systematic review and meta-analysis

**DOI:** 10.1007/s00520-025-09667-4

**Published:** 2025-06-30

**Authors:** Xiaotian Huang, Kefu Shi, Norhasmah Mohd Zain, Azlina Yusuf

**Affiliations:** 1 Nursing Programme, School of Health Sciences, Health Campus, Universiti Sains Malaysia, Kota Bharu, Kelantan Malaysia; 2 Nursing Department, Shenzhen Qianhai Taikang Hospital, No. 3099 Menghai Avenue, Nanshan District, Shenzhen, Guangdong Province People’s Republic of China 518000; 3 Department of Radiation Oncology, State Key Laboratory of Oncology in South China, Collaborative Innovation Center for Cancer Medicine, Guangdong Key Laboratory of Nasopharyngeal Carcinoma Diagnosis and Therapy, Sun Yat-sen University Cancer Center, Guangzhou, Guangdong People’s Republic of China 510000

**Keywords:** Cancer patients, Peripherally inserted central catheters (PICCs), Home care-based education, Continuity of care, Meta-analysis, Self-management

## Abstract

**Purpose:**

This study aimed to systematically review and meta-analyze the literature to identify the components of home care-based education for cancer patients with peripherally inserted central catheters (PICCs) and evaluate its effects compared to traditional health education.

**Methods:**

A comprehensive search was conducted in Chinese and English databases until November 2024. The searching strategy, screening, quality assessment data extraction, and meta-analysis were performed scientifically.

**Results:**

A total of 19 studies were included. Three core components of home care-based education were identified: catheter care, self-care, and resource support. Meta-analysis revealed significant improvements in eight outcomes for intervention groups: improved self-management abilities (*MD* = 17.77, 95% *CI*, *Z* = 9.2,* P* < 0.00001); reduced anxiety (*MD* = −8.53, 95% *CI*, *Z* = 4.56, *P* < 0.00001) and depression (*MD* = −11, 95% *CI*, *Z* = 3.97, *P* < 0.0001); lower complication incidence (*OR* = 0.17, 95% *CI*, *Z* = 11.79, *P* < 0.00001); higher compliance rate (*OR* = 0.16, 95% *CI*, *Z* = 4.98, *P* < 0.00001); improved self-efficacy (*MD* = 9.45, 95% *CI*, *Z* = 2.41, *P* = 0.02); increased satisfaction with nursing care (*OR* = 6.01, 95% *CI*, *Z* = 6.19, *P* < 0.00001); better quality of life (*MD* = 9.38, 95% *CI*, *Z* = 4.39, *P* < 0.0001).

**Conclusions:**

Home care-based education improves self-management, psychological well-being, treatment outcomes, and satisfaction in cancer patients with PICCs. The identified components provide a practical framework for clinical implementation, though cultural adaptability and protocol standardization require further study.

Trial registration

CRD42024606607

## Introduction

The predominant approaches employed in the treatment of cancer presently encompass three primary modalities, namely chemotherapy, radiotherapy, and surgery [1]. In situations where cancer has extended beyond its primary location, the administration of chemotherapy may be advised [2]. Peripherally inserted central catheters (PICCs) play a significant role in the chemotherapy of cancer, primarily due to their suitability for long-term intravenous therapy [3]. There are many advantages to PICCs. After inserting PICCs, it can reduce the frequency of needle sticks for patients, which reduces the discomfort and stress of repeated venipuncture for patients [4]. PICCs are essential for the administration of chemotherapy drugs. These drugs can be harsh on smaller, peripheral veins, but PICC lines, which extend to a large central vein near the heart, are more durable and less likely to cause vein irritation or damage [5]. Besides chemotherapy, cancer patients may need various other medications, such as antibiotics, pain relievers, antiemetics [6] even total parenteral nutrition (TPN) [7]. PICCs allow for these medications to be administered directly into the bloodstream, ensuring rapid and efficient absorption [6]. While there is an infection risk with central lines, PICCs are generally associated with a lower risk compared to other types of central venous catheters [8].

The trend now is to encourage cancer patients to practice self-care at home and reduce hospital stays [9]. Cancer patients often experience significant stress due to their illness and treatment regimens, and receiving care at home can alleviate some of this by providing a more comfortable and familiar environment [10]. Besides, considering the economic and social needs of patients and the consumption of medical resources, patients are advised to be discharged from the hospital and go home to recuperate when they have a stable health condition, and wait for the next cycle of chemotherapy [11].

Therefore, the questions that guided the systematic review were as follows.

1. What is the component of home care-based education on PICC for cancer patients?
2. Is the home care-based education program for cancer patients with PICC more effective compared to traditional health education?

## Methods

The protocol of this systematic review and meta-analysis was registered with PROSPERO on November 6, 2024, number CRD42024606607. The ethical approval was not necessary and was waived.

### Search strategy

The researchers used the keywords “peripherally inserted central catheter* OR PICC* OR vascular access device* OR peripheral catheter* OR peripheral intravenous catheter,” “cancer OR carcinoma OR malignant tumour* OR neoplas* OR malignant neoplasm OR malignanc*,” “health education OR patient education OR home care-based education OR self-care OR continuing nursing service” to conduct the searching in the Chinese databases, including CNKI, VIP database and Wanfang database, and English databases, including PubMed, Scopus, Web of Science, and The Cochrane Library. The search period was from the establishment of the database to November 2024. Additionally, a secondary search was conducted by thoroughly reading relevant literature. The searching strategies of different databases are shown in Table 1.

**Table 1:** Searching strategy

Database	Searching strategy
Pubmed	#1 ((((peripherally inserted central catheter*[MeSH Terms]) OR (PICC*)) OR (vascular access device*)) OR (peripheral catheteri*)) OR (peripheral intravenous catheter)
	#2 (((((cancer[MeSH Terms]) OR (carcinoma)) OR (malignant tumour*)) OR (neoplas*)) OR (malignant neoplasm)) OR (malignanc*)
	#3 ((((health education) OR (patient education)) OR (home care-based education)) OR (self-care)) OR (continuing nursing service)
	#1 AND #2 AND #3
Scopus	1. (TITLE-ABS-KEY (peripherally AND inserted AND central AND catheter*) OR TITLE-ABS-KEY (picc*) OR TITLE-ABS-KEY (vascular AND access AND device*) OR TITLE-ABS-KEY (peripheral AND catheteri*) OR TITLE-ABS-KEY (peripheral AND intravenous AND catheter))
	(TITLE-ABS-KEY (cancer) OR TITLE-ABS-KEY (carcinoma) OR TITLE-ABS-KEY (malignant AND tumour*) OR TITLE-ABS-KEY (neoplas*) OR TITLE-ABS-KEY (malignant AND neoplasm) OR TITLE-ABS-KEY (malignanc*))
	(TITLE-ABS-KEY (health AND education) OR TITLE-ABS-KEY (patient AND education) OR TITLE-ABS-KEY (home AND care-based AND education) OR TITLE-ABS-KEY (self-care) OR TITLE-ABS-KEY (continuing AND nursing AND service))
	((TITLE-ABS-KEY (health AND education) OR TITLE-ABS-KEY (patient AND education) OR TITLE-ABS-KEY (home AND care-based AND education) OR TITLE-ABS-KEY (self-care) OR TITLE-ABS-KEY (continuing AND nursing AND service))) AND ((TITLE-ABS-KEY (cancer) OR TITLE-ABS-KEY (carcinoma) OR TITLE-ABS-KEY (malignant AND tumour*) OR TITLE-ABS-KEY (neoplas*) OR TITLE-ABS-KEY (malignant AND neoplasm) OR TITLE-ABS-KEY (malignanc*))) AND ((TITLE-ABS-KEY (peripherally AND inserted AND central AND catheter*) OR TITLE-ABS-KEY (picc*) OR TITLE-ABS-KEY (vascular AND access AND device*) OR TITLE-ABS-KEY (peripheral AND catheteri*) OR TITLE-ABS-KEY (peripheral AND intravenous AND catheter)))
Web of Science	#1 TS=(peripherally inserted central catheter*) OR TS=(PICC*) OR TS=(vascular access device*) OR TS=(peripheral catheteri*) OR TS=(peripheral intravenous catheter)
	#2 TS=(cancer) OR TS=(carcinoma) OR TS=(malignant tumour*) OR TS=(neoplas*) OR TS=(malignant neoplasm) OR TS=(malignanc*)
	#3 TS=(health education) OR TS=(health education) OR TS=(home care-based education) OR TS=(self-care) OR TS=(continuing nursing service)
	#1 AND #2 AND #3
The Cochrane Library	#1 (peripherally inserted central catheter):ti,ab,kw OR (PICC):ti,ab,kw OR (vascular access device):ti,ab,kw OR (peripheral catheteri*):ti,ab,kw OR (eripheral intravenous catheter):ti,ab,kw (Word variations have been searched)
	#2 (cancer):ti,ab,kw OR (carcinoma):ti,ab,kw OR (malignant tumour*):ti,ab,kw OR (malignant neoplasm):ti,ab,kw OR (malignanc*):ti,ab,kw (Word variations have been searched)
	#3 (health education):ti,ab,kw OR (patient education):ti,ab,kw OR (home care-based education):ti,ab,kw OR (self-care):ti,ab,kw OR (continuing nursing service):ti,ab,kw (Word variations have been searched)
	#1 AND #2 AND #3

### Eligibility criteria

Inclusion criteria: ① the study type is clinical research, ② the subjects are ≥18 years old and diagnosed with malignant tumors and carry PICC, ③ the research intervention is health education for cancer patients, ④ the literature fully reports the research findings.

Exclusion criteria: ① the language is non-Chinese and non-English, and there is no translation, ② duplicate literature, ③ the article type is review, evaluation, conference proceedings, and animal research.

### Literature screening and data extraction

This study was carried out by a research team consisting of three researchers. Two researchers read and screened all the titles and abstracts of the literature in accordance with the eligibility criteria. Only full-text articles were retrieved from the literature that met the inclusion criteria. Suppose it was impossible to determine whether to include a piece of literature based on the abstract data, the full text was downloaded for further assessment. In case of disagreement, it would be resolved through consensus with the third researcher. The relevant data were extracted in the form of electronic data. The extracted information encompassed: ① Basic information of the included literature, such as the year of publication, research field, and research subjects; ② Basic information of the research subjects, including region; ③ Information on the intervention experiments, such as sample size, intervention measures, intervention duration, duration of intervention, delivery method, and educator qualifications; ④ Education components and contents each study conducted; ⑤ Outcome indicators, including patients’ self-care ability, quality of life, mental status, and the incidence of complications.

### Literature quality evaluation

Different types of research employ distinct quality assessment tools. This study encompasses randomized controlled trials, cohort studies, and qualitative research. Consequently, corresponding and disparate quality assessment tools will be utilized to evaluate their quality.

The Cochrane Risk of Bias tool [12] was employed to assess randomized controlled trials. This instrument specifically addresses potential biases in several critical domains, including those introduced during the randomization process, deviations from intended interventions, missing outcome data, the measurement of outcomes, and the selection of reported outcomes. Comprising six key domains, the Cochrane Risk of Bias tool allows reviewers to evaluate the risk of bias in each area, categorizing it as “low risk,” “unclear risk,” or “high risk.” These evaluations contribute to an overall judgment regarding the risk of bias for each study, ensuring a rigorous and systematic assessment of study quality.

The JBI (Joanna Briggs Institute) quasi-experimental research literature quality assessment tool was utilized to assess quasi-experimental studies in the systematic review [13]. This tool specifically focuses on potential quality issues in several important aspects, including the design and methodology of the study, the collection and measurement of data, and the analysis and reporting of results. Comprising multiple specific criteria, the JBI tool enables reviewers to evaluate the quality in each area by answering nine questions, categorizing it as a score of “2” (satisfactory), “1” (needs improvement), or “0” (unsatisfactory).

The CASP checklist was used to assess the quality of qualitative study [14], which contains three sections ten questions. The reviewers assessed the articles by answering each question with “yes,” “no,” or “can’t tell.”

### Data synthesis and statistical analysis

EndNote X8 software was utilized to eliminate duplicates and synthesize the literature systematically. Data extraction, organization, and descriptive analysis were performed using Excel, ensuring a structured approach to data management. For statistical analyses, RevMan 5.3 software was employed, with odds ratio (*OR*) values and *95%* confidence intervals (*CI*) serving as indicators of statistical effect size [15]. The heterogeneity among studies was assessed using the* I*^*2*^ statistic [16]. When* P* > 0.10 and *I*^*2*^ < 50%, heterogeneity was considered acceptable, allowing for the application of a fixed effects model. Conversely, when *P* ≤ 0.10 and *I*^*2*^ ≥ 50%, substantial heterogeneity was inferred, necessitating using a random effects model. In cases where the literature consisted of fewer than two studies, a descriptive analysis was conducted. To address potential biases, sensitivity analyses and subgroup analyses were performed where appropriate, aiming to assess the robustness of the findings and identify sources of variability, thereby enhancing the reliability of the meta-analysis outcomes. To address potential publication bias, funnel plots were generated for main outcomes with ≥10 studies to visually assess asymmetry, and Egger’s linear regression test was performed using STATA 17.0. A significance level of *P* < 0.05 indicated potential publication bias.

## Results

### Literature selection

**Fig. 1:**
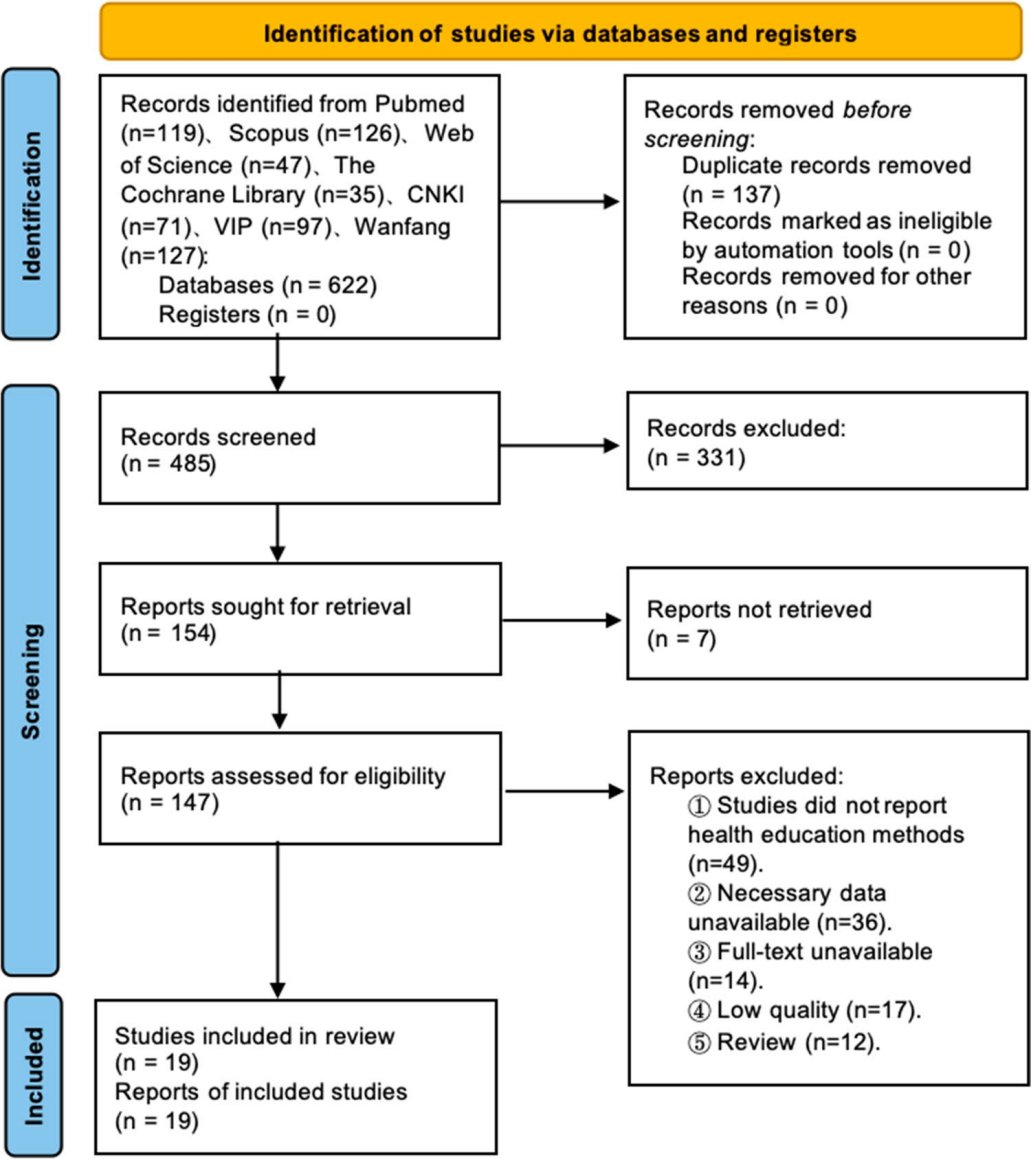
Flow diagram of the systematic review

A total of 622 studies were obtained after the initial search. After eliminating duplicates, 485 studies remained. After the title and abstract screening, 331 records were excluded. Subsequently, 154 reports were sought for retrieval, and seven reports were not retrieved. A total of 147 studies remain to be assessed for eligibility. In this process, 48 studies were excluded because they did not report health education methods, 38 studies were excluded due to necessary data being unavailable, 14 studies were excluded because the full texts were unavailable, 17 studies were excluded because of low quality, and 12 studies were excluded because they were reviews. Finally, 19 studies were included in the synthesis. Figure 1 shows the flow diagram of the study selection.

### Study characteristics

All the included studies were published between 2021 and 2024. A total of 3043 patients with PICC catheterization were included in the 19 studies, with 15–329 patients in each study. Among the included studies, 18 studies were from Asian countries (China) [11, 17–33], and one from Australia [34]. The study designs included 14 randomized controlled trials [11, 17, 19, 20, 22–24, 26–31, 33], four quasi-experimental studies [18, 21, 25, 32], and one qualitative study [34]. The quality assessment was conducted by using the Cochrane risk of bias for RCTs, the Joanna Briggs Institute (JBI) quasi-experimental research literature quality assessment tool for quasi-experimental studies, and the Critical Appraisal Skills Programme (CASP) tool for qualitative study.

Table 2 presents the findings and data extractions of the studies.

**Table 2:** Findings and data extractions of review studies

No.	Author/year	Region	Study design	Sample size	Intervention				Education contents	Improved outcome indicators
					Contents	Duration	Delivery method	Educators qualification		
1.	Zhu et al., 2021 [11]	China (Xinjiang)	Randomized controlled trial	90 (45 in each group)	Observation group: Continued nursing service including a continuous nursing team, whole-course tracking system, inpatient care, post-catheterization care, discharge instructions, outpatient maintenance, and mutual-help groups; Control group: Conventional nursing service.	3 months	Face-to-face education + telephone follow-up	PICC-certified nurses	Catheter maintenance, self-management, family education, medication guidance.	Self-management ability (ESCA score), catheter indwelling time, on-time maintenance ratio, compliance, complication rate and nursing satisfaction.
2.	Zhang et al., 2021 [17]	China (Beijing)	Randomized controlled trial	160 (80 in each group)	Observation group: PICC specialist nursing and continuous self-management education with an education team and inpatient/outpatient education; control group: conventional care (return to original catheterization hospital or catheter maintenance in nursing clinic).	3 months	Face-to-face education + telephone follow-up	Senior nurses, head nurses	Basic information on PICC, catheter maintenance, self-management, and regular review reminders are needed.	Self-management ability (ESCA score), health behavior score, complications.
3.	Wang et al., 2021 [18]	China (Hainan)	Quasi-experimental study	120 (60 in each group)	Observation group: health education including pre-, during-, and post-catheterization guidance in addition to conventional nursing; control group: conventional nursing.	3 months	WeChat + face-to-face education	PICC-qualified nurses, oncologists	Basic information on PICC, catheterization process, catheter maintenance, and precautions of the emergencies.	Health promotion lifestyle (HPLP-II score), self-efficacy (CPPSM score), anxiety and depression status (SAS and SDS scores), maintenance compliance rate, catheter placement complication rate, PICC maintenance failures.
4.	Wan, 2022 [19]	China (Henan)	Randomized controlled trial	100 (50 in each group)	Observation group: health education training in addition to conventional nursing; control group: conventional nursing.	3 months	Face-to-face education + telephone follow-up	Healthcare professionals	Information on catheter maintenance and self-management.	Catheter maintenance compliance, anxiety, and depression status (SAS and SDS scores), PICC maintenance failures, complication rate.
5.	Huang et al., 2023 [20]	China (Jiangsu)	Randomized controlled trial	160 (80 in each group)	Observation group: “Hospital - Community - Family” tertiary linkage nursing model; Control group: Conventional nursing and education.	3 months	Home visits, community nurse follow-ups	PICC-certified nurses, social workers	Follow-up guidance and home care guidance.	Compliance, complication rate, negative emotions, quality of life.
6.	Gao et al., 2024 [21]	China (Shaanxi)	Quasi-experimental study	131 (70 in the observation group and 61 in the control group)	Observation group: Internet-based multidisciplinary continuous nursing with a WeChat public account and a multidisciplinary continuous nursing team; control group: conventional discharge guidance and telephone follow-up.	6 months	WeChat	Specialist physicians, nutritionists, psychologists, senior nurses	PICC-related knowledge, catheter maintenance methods, FAQs, personalized nursing guidance involving psychological, nutritional, and other knowledge.	Self-management (ESCA score), quality of life, treatment compliance, complication rate, and nursing satisfaction.
7.	Tao et al., 2023 [22]	China (Heilongjiang)	Randomized controlled trial	96 (48 in the each group)	Observation group: health education intervention with the teach-back method; control group: routine care	3 months	Face-to-face education	Head nurses, PICC-certified nurses, physicians	Precautions during the catheter indwelling period, observation and treatment of catheter abnormalities, home activity range, self-observation of catheter abnormalities and catheter self-maintenance.	Mental state (Hospital Anxiety and Depression Scale), incidence of complications, quality of life (Short Form Health Survey).
8.	Zhang et al., 2023 [23]	China (Fujian)	Randomized controlled trial	130 (65 in the each group)	Observation group: “Internet + nursing health education” nursing model Control group: Routine PICC catheterization nursing model	6 months	WeChat	Oncology directors, head nurses, associate chief nurses	PICC nursing-related knowledge, self-management methods, psychological counseling.	Anxiety and depression status (SAS and SDS scores), maintenance compliance, complication incidence, and medical staff satisfaction.
9.	Zhang et al., 2022 [24]	China (Henan)	Randomized controlled trial	78 (39 in each group)	Observation group: Health education based on the empowerment workshop Control group: Routine health education	1 month	Face-to-face Empowerment workshop	Senior nurses	Introduction of PICC, cooperation and precautions, self-maintenance method and complications notification.	Anxiety and depression status (SAS and SDS scores), self-efficacy (General Self-Efficacy Scale), self-management ability (ESCA Scale), and coping style (Medical Coping Style Questionnaire).
10.	Lu et al., 2024 [25]	China (Jiangsu)	Quasi-experimental study	118 (58 in the observation group and 60 in the control group)	Observation group: WeChat-based continuous nursing with mind map health education Control group: Routine continuous nursing	3 months	WeChat + Mind maps	Intravenous therapy specialist nurses	Daily catheter observation, life with a catheter, information acquisition, management confidence, abnormal handling, maintenance compliance, and exercise with a catheter.	Catheter maintenance quality, complication incidence, health education Observationeffect, self-management ability (ESCA Scale).
11.	Zhou, 2023 [26]	China (Jiangsu)	Randomized controlled trial	80 (40 in each group)	Observation group: Health education based on the WeChat platform Control group: Routine health education	1 month	WeChat	Continuity nursing team members	Introduction of PICC; procedure cooperation method; catheter maintenance methods, testing methods.	Self-management ability, quality of life, complications.
12.	Jiang et al., 2022 [27]	China (Shanghai)	Randomized controlled trial	329 (165 in the observation group and 164 in the control group)	Observation group: Health education based on the ADOPT model, which includes five aspects: Attitude, Definition, Open mind, Planning, and Trying it out. Control group: Routine health education methods	6 months	Face-to-face education	PICC-certified nurses, intravenous infusion committee members	Introduction of PICC, precautions, self-maintainers methods, education on patients’ family members.,	Self-management ability, complication incidence, outpatient catheterized patient satisfaction rate.
13.	Zhong et al., 2023 [28]	China (Anhui)	Randomized controlled trial	64 (32 in each group)	Observation group: WeChat combined with the teach-back method Control group: Routine health education methods for PICC catheterization	3 months	WeChat + Telephone follow-up	Health education nurses	Information on PICC and catheter maintenance methods.	Awareness rate of educational content, complication incidence.
14.	Liu and Yang, 2024 [29]	China (Jiangxi)	Randomized controlled trial	60 (30 in each group)	Observation group: Systematic health education intervention Control group: Routine nursing intervention	4 weeks	Face-to-face education + WeChat follow-up	Physicians, PICC-certified nurses	PICC-related knowledge, catheter maintenance methods.	Self-management ability (ESCA Scale), Self-efficacy (CPPSM score), compliance rate, and complication incidence.
15.	Wang, 2023 [30]	China (Liaoning)	Randomized controlled trial	70 (35 in each group)	Observation group: Information nursing health education combined with structural psychological intervention Control group: Routine intervention	1 month	Face-to-face education + Telephone	Senior nurses	Introduction of PICC, cooperation of PICC procedure, the self-management methods of PICC, including diet, bath, and exercise.	Health knowledge mastery (Health Knowledge Mastery Scale), mental state (Mental State Scale), complications, and satisfaction.
16.	Lu, 2024 [31]	China (Fujian)	Randomized controlled trial	166 (83 in each group)	Observation group: Continuity of care Control group: Routine care	3-month follow-up	WeChat	Head nurses	PICC-related knowledge, catheter maintenance methods.	Complication incidence, self-management ability, quality of life (Functional Assessment of Cancer Therapy - General Module Scale).
17.	Wu, 2024 [32]	China (Zhejiang)	Quasi-experimental study	120 (60 in each group)	Observation group: KTH integrated health education. combined with the knowledge, belief and practice health education model, a health education group was established to provide patients with personalized and diversified health education forms. Control group: Routine propaganda methods	3 months	WeChat + workshop	PICC-qualified nurses, social workers, psychologists, nutritionists	Introduction of PICC, the self-management methods of PICC, including diet, psychological care, hospital and family support.	Self-management ability, caregiver satisfaction, complications, quality of life (Quality of Life Scale).
18.	Zhou and Wu, 2023 [33]	China (Fujian)	Randomized controlled trial	60 (30 in each group)	Observation group: Health education and the teach-back method Control group: Routine health education method	3 months	Face-to-face education	Senior nurses	PICC-related knowledge, self-management methods.	Self-management ability (Patient PICC Self-Management Ability Scale)
19.	Sharp et al., 2024 [34]	Australia	qualitative study	15	Interviews to understand supportive care needs.				Guidance on how to manage daily activities, information about the PICC insertion process, guidance on self-monitoring for complications, clear Communication of Responsibility, and supportive Resources.	

**Fig. 2:**
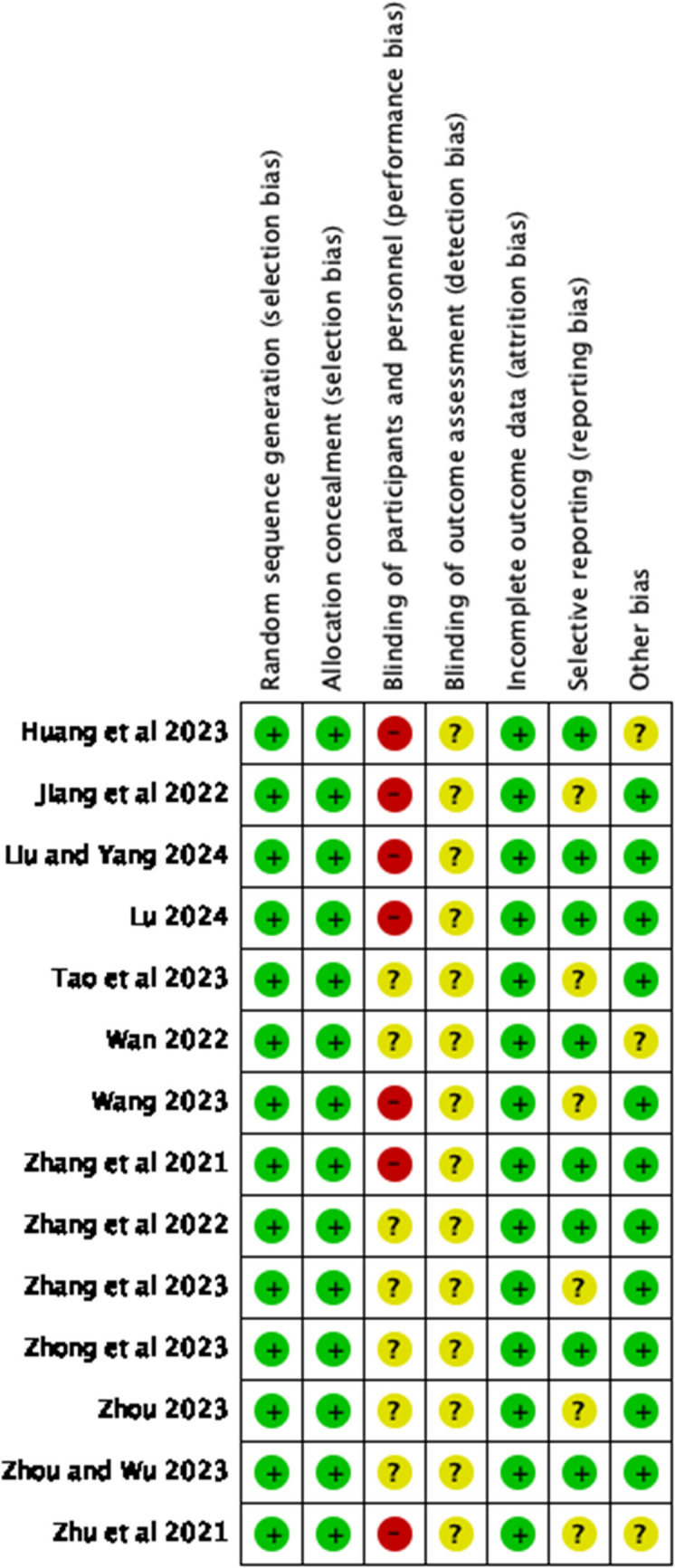
Risk of bias summary

**Fig. 3:**
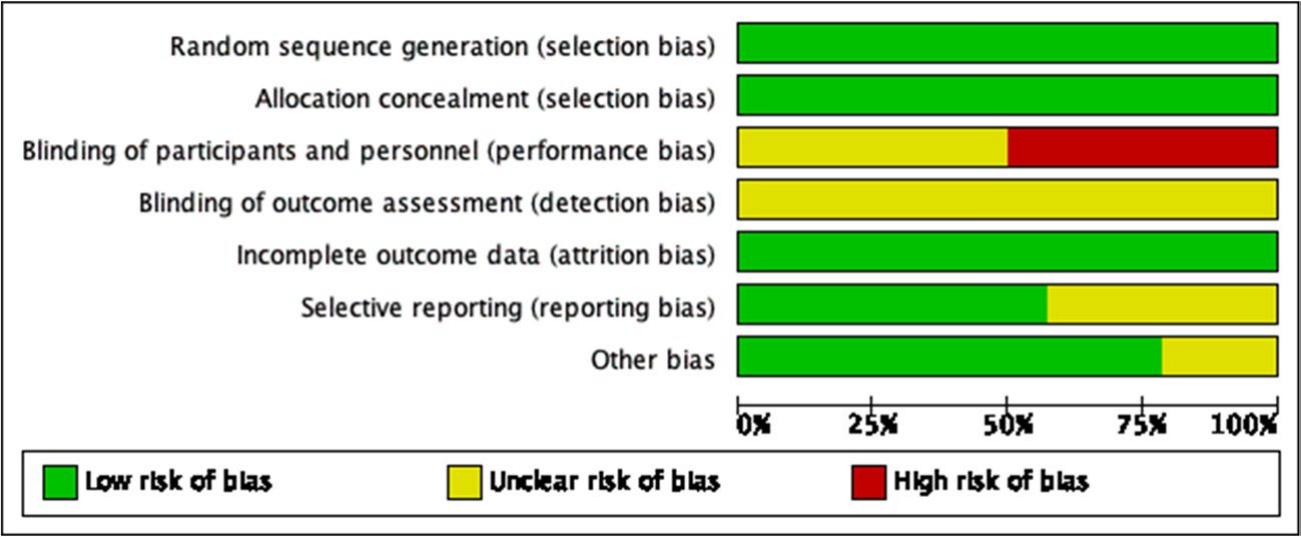
Risk of bias graph

The specific criteria used for evaluating the quality of RCTs are outlined in Figs. 2 and 3. The details of quasi-experimental study assessment are shown in Table 3. The assessment of the qualitative study is shown in Table 4. According to the RoB 2 assessment, all included randomized controlled trials (RCTs) demonstrated low risk of selection bias and attrition bias (100% of studies). Over 50% of studies (7/14) exhibited low risk in reporting bias, with other bias categories (e.g., confounding) also showing low-risk rates exceeding 75%. However, performance bias was rated as a moderate risk in 50% (7/14) and high risk in 50% (7/14) of studies, primarily due to inadequate reporting on the blinding of participants/personnel (12/14 studies lacked explicit descriptions). Furthermore, detection bias was uniformly classified as moderate risk (14/14 studies), as none implemented blinding of outcome assessment. The four quasi-experimental studies [18, 21, 25, 32] assessed by the JBI tool demonstrated that 75% (3/4) achieved a “satisfactory” quality rating (total score ≥14/18). All articles did not provide detailed information on the consistency of other interventions (such as nutritional support and psychological counseling) between the intervention and control groups, so this aspect was marked as “needs improvement.” Moreover, 75% (3/4) failed to analyze differences in dropout data, potentially introducing selection bias. The single qualitative study [34] evaluated using the CASP tool, excelled in clarity of research aims, methodological appropriateness, and ethical rigor. Nevertheless, researchers did not reflect on their roles in shaping outcomes, and the exclusively Australian sample limited cross-cultural generalizability. Overall, all the articles have good quality, but still have some problems that may lead to bias.

**Table 3:** Assessment of quasi-experimental studies by using The JBI (Joanna Briggs Institute) quasi-experimental research literature quality assessment tool

Articles	Question No. 1	Question No. 2	Question No. 3	Question No. 4	Question No. 5	Question No. 6	Question No. 7	Question No. 8	Question No. 9	Total scores
Wang et al., 2021 [18]	2	2	1	2	2	1	2	2	2	16
Gao et al., 2024 [21]	2	2	1	2	2	1	2	2	2	16
Lu et al., 2024 [25]	2	2	1	2	1	1	2	2	2	15
Wu, 2024 [32]	2	2	1	2	2	2	2	2	2	17

The questions of The JBI (Joanna Briggs Institute) quasi-experimental research literature quality assessment tool are as follows:

1. Is it clear in the study what is the “cause” and what is the “effect” (i.e., there is no confusion about which variable comes first)?

2. Were the participants included in any comparisons similar?

3. Were the participants included in any comparisons receiving similar treatment/care, other than the exposure or intervention of interest?

4. Was there a control group?

5. Were there multiple measurements of the outcome both pre and post the intervention/exposure?

6. Was follow-up complete and if not, were differences between groups in terms of their follow up adequately described and analyzed?

7. Were the outcomes of participants included in any comparisons measured in the same way?

8. Were outcomes measured in a reliable way?

9. Was appropriate statistical analysis used?

**Table 4:** Assessment of CASP checklist for qualitative study

Section A Are the results valid?	
1. Was there a clear statement of the aims of the research?	Yes, to identify the supportive care needs.
2. Is a qualitative methodology appropriate?	Yes, using semi-structured interviews.
3. Was the research design appropriate to address the aims of the research?	Yes, the qualitative description was used.
4. Was the recruitment strategy appropriate to the aims of the research?	Yes, A purposeful sampling approach was used.
5. Was the data collected in a way that addressed the research issue?	Yes, semi-structured telephone or video interviews were conducted the results can guide further exploration.
6. Has the relationship between researcher and participants been adequately considered?	Yes, a process was in place to obtain informed consent in an appropriate way to avoid influencing participants’ ability to provide voluntary consent.
Section B: What are the results?	
7. Have ethical issues been taken into consideration?	Yes, ethical approval was obtained prior to the study’s commencement, and informed consent was obtained from participants.
8. Was the data analysis sufficiently rigorous?	Yes, interviews were transcribed, and a qualitative content analysis technique was used to analyze both manifest and latent content. Two researchers independently read transcripts and compared coding, and data saturation was determined.
9. Is there a clear statement of findings?	Yes, participants’ supportive care needs are determined.
Section C: Will the results help locally?	
10. How valuable is the research?	Yes, the research is valuable as it provides insights into the supportive care needs of adults living with a PICC at home, which can inform nursing assessment and the provision of appropriate support. It fills a gap in the research on this topic.

**Fig. 4:**
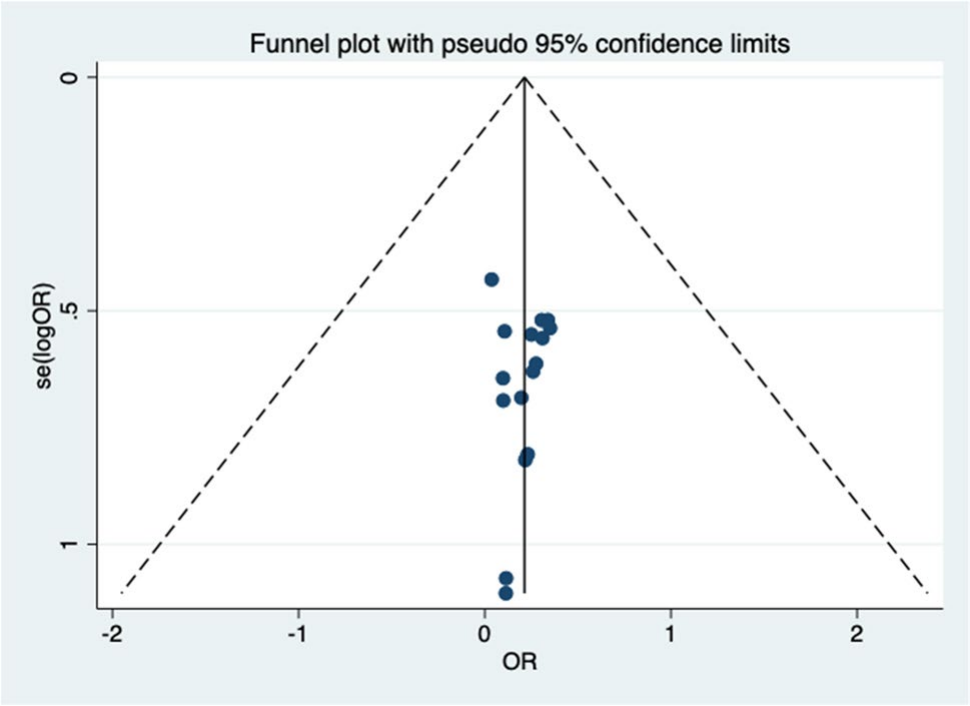
Funnel plot

Funnel plots and Egger’s tests were conducted for main outcomes with sufficient studies (*n* ≥ 10). For complication incidence (*n* = 16 studies), visual inspection of the left and right sides of the funnel plot is relatively symmetrical (Fig. 4). Egger’s test confirmed no significant publication bias (*t* = −0.25, *p* = 0.804).

### Results of the education components

#### Catheter care

Fifteen articles [17, 18, 21–24, 26–34] reported that catheter care education is one of the components of PICC home care-based education, including the introduction of PICC, introduction to the catheter insertion process, and precautions for the catheter insertion period.

#### Self-care

All the 19 articles [11, 17–34] reported that providing self-care education to the patients is a crucial component of conducting PICC home care-based education, including catheter management methods and self-management methods. Among them, five articles [21, 25, 30, 32, 34] emphasized that when patients conduct self-care, they need to pay attention to daily activities including diet, bath, and exercise. Three articles [17, 18, 25] underlined that educating patients on emergency precautions and handling is a vital aspect of patient self-care. Three articles [22, 24, 34] highlighted the significance of patient education on catheter self-observation and complication prevention as a crucial aspect of self-care.

#### Resource support

Six articles [11, 21, 23, 27, 32, 34] indicated that incorporating resources for support from both home and hospital settings is essential for home care-based education on patients with PICC. Two of the articles [21, 23] underscored the significance of providing psychological support.

### Meta-analysis

#### Self-management abilities

**Fig. 5:**
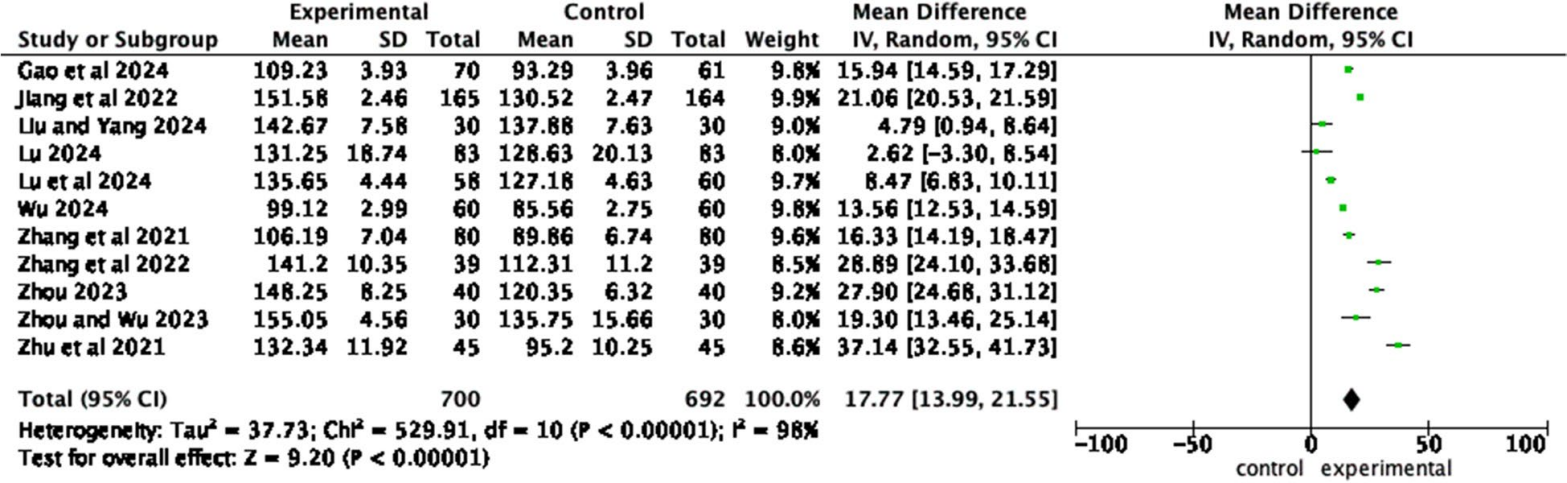
Forest plot of the self-management abilities

Eleven articles [11, 17, 21, 24–27, 29, 31–33] reported the self-management abilities of the observation group and the control group. The results of the heterogeneity test showed *I*^2^ = 98%, *P* < 0.00001, and a random effects model was employed. Meta-analysis results show that compared with patients who received routine health education, patients who received home care-based education had significantly enhanced self-management abilities (*MD* = 17.77, 95% *CI*, *Z* = 9.2, *P* < 0.00001) (Fig. 5).

#### Mental status

**Fig. 6:**
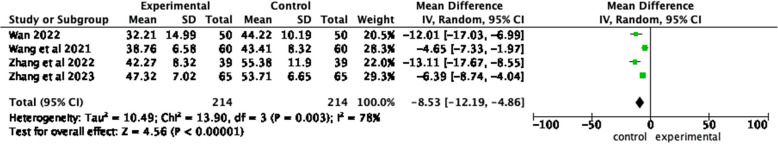
Forest plot of anxiety status

Five articles [18–20, 23, 24] reported the anxiety and depression status between the observation group and the control group measured by SAS and SDS. One of the articles [19] did not provide continuous variables data, leading to its exclusion from the meta-analysis. In the meta-analysis of anxiety status, the results of the heterogeneity test showed *I*^2^ = 78%, *P* = 0.003, and a random effects model was employed. Meta-analysis results show that compared with patients who received routine health education, patients who received home care-based education had lower anxiety incidence (*MD* = −8.53, 95% *CI*, *Z* = 4.56, *P* < 0.00001) (Fig. 6).

**Fig. 7:**
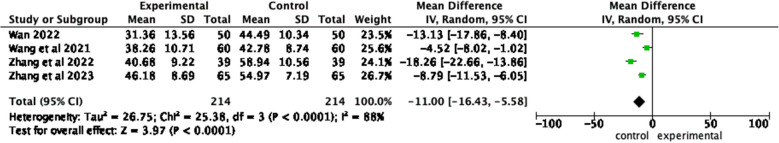
Forest plot of depression status

In the meta-analysis of depression status, the results of the heterogeneity test showed *I*^2^ = 88%, *P* < 0.0001, and a random effects model was employed. Meta-analysis results show that compared with patients who received routine health education, patients who received home care-based education had lower depression incidence (*MD* = −11, 95% *CI*, *Z* = 3.97, *P* < 0.0001) (Fig. 7).

#### Complication incidence

**Fig. 8:**
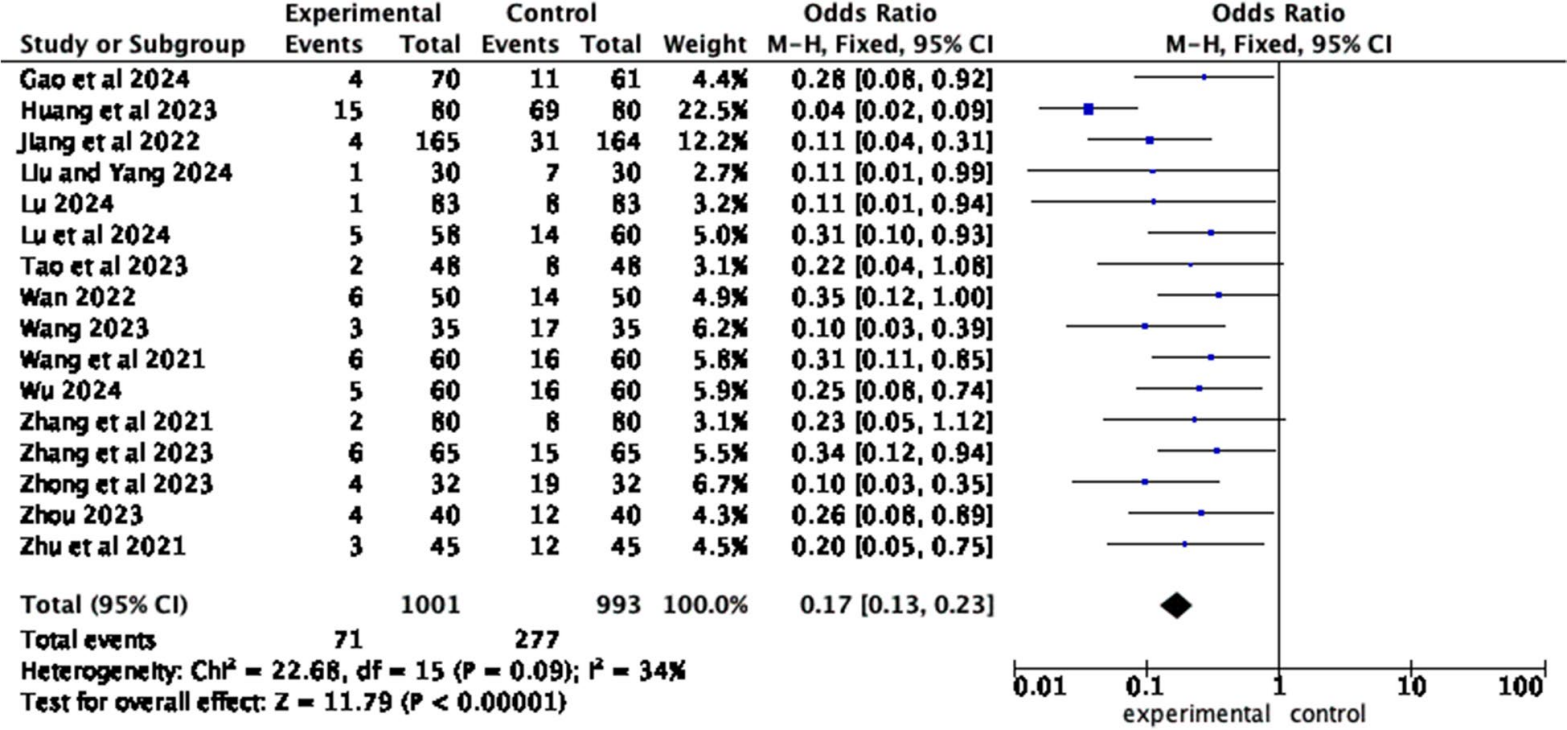
Forest plot of complication incidence

Sixteen articles [11, 17–23, 25–32] reported the complication incidence between the observation group and the control group. The results of the heterogeneity test showed *I*^2^ = 34%, *P = 0.09*, and a fixed effects model was employed. Meta-analysis results show that compared with patients who received routine health education, patients who received home care-based education had lower complication incidence (*OR* = 0.17, 95% *CI*, *Z* = 11.79, *P* < 0.00001) (Fig. 8).

#### Compliance

**Fig. 9:**
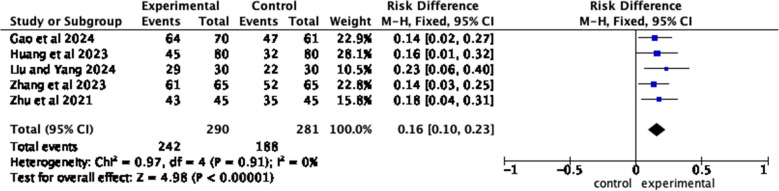
Forest plot of compliance rate

Five articles [11, 20, 21, 23, 29] reported the compliance between the observation group and the control group. The results of the heterogeneity test showed *I*^2^ = 0%, *P = 0.91*, and a fixed effects model was employed. Meta-analysis results show that compared with patients who received routine health education, patients who received home care-based education had a higher compliance rate (*OR* = 0.16, 95% *CI*, *Z* = 4.98, *P* < 0.00001) (Fig. 9).

#### Self-efficacy

**Fig. 10:**
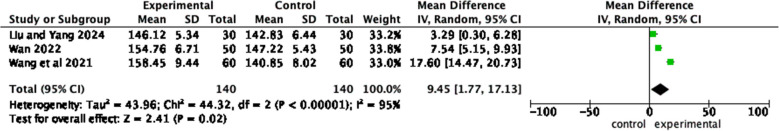
Forest plot of self-efficacy

Four articles [18, 19, 24, 29] reported the self-efficacy between the observation group and the control group. Three of them were measured by CPPSM scores [18, 19, 29], and one was measured by GSES scores [24], which led to its discard from this meta-analysis. The results of the heterogeneity test showed *I*^2^ = 95%, *P* < 0.00001, and a random effects model was employed. Meta-analysis results show that compared with patients who received routine health education, patients who received home care-based education had a higher self-efficacy rate (*MD* = 9.45, 95% *CI*, *Z* = 2.41, *P =* 0.02) (Fig. 10).

#### Satisfaction

**Fig. 11:**
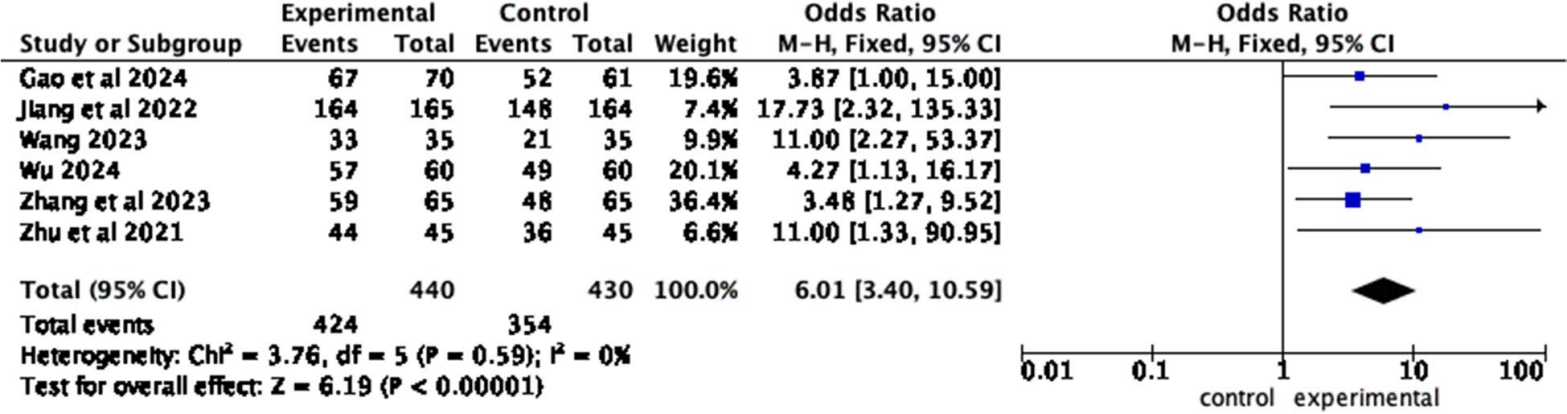
Forest plot of satisfaction

Six articles [11, 21, 23, 27, 30, 32] reported patient satisfaction between the observation group and the control group. The results of the heterogeneity test showed *I*^2^ = 0%, *P =* 0.59, and a fixed effects model was employed. Meta-analysis results show that compared with patients who received routine health education, patients who received home care-based education had higher satisfaction with nursing care service (*OR* = 6.01, 95% *CI*, *Z* = 6.19, *P* < 0.00001) (Fig. 11).

#### Quality of life

**Fig. 12:**
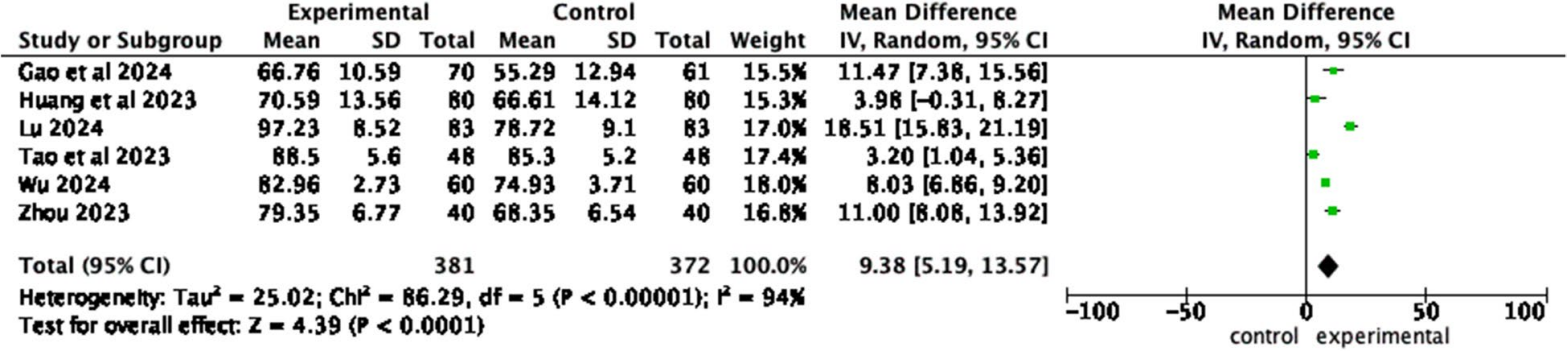
Forest plot of quality of life

Six articles [20–22, 26, 31, 32] reported the quality of life between the observation group and the control group. The results of the heterogeneity test showed *I*^2^ = 94%, *P* < 0.00001, and a random effects model was employed. Meta-analysis results show that compared with patients who received routine health education, patients who received home care-based education had better quality of life (*MD* = 9.38, 95% *CI*, *Z* = 4.39, *P* < 0.0001) (Fig. 12).

In summary, this study revealed the significant effect of home care-based education on eight key outcome indicators through meta-analysis. The significant improvement in self-management ability (*MD* = 17.77, 95% CI 13.99–21.55) and quality of life (*MD* = 9.38, 95% CI 5.19–13.57) indicated that the intervention measures could effectively enhance the patients’ autonomous care ability and overall health status. In terms of psychological state, the negative effect sizes of anxiety (*MD* = −8.53) and depression (*MD* = −11) showed that the emotional distress in the intervention group was significantly reduced, suggesting that home care-based education played an important role in improving the psychological stress of cancer patients. The odds ratios of complication incidence (*OR* = 0.17) and treatment compliance (*OR* = 0.16) showed that home care-based education could reduce the risk of complications by 83% and improve treatment compliance by 84%. The significant improvement in patient satisfaction (*OR* = 6.01) further verified the value of home care-based education in optimizing the medical experience. It is worth noting that the improvement of self-efficacy (*MD* = 9.45) provides psychological support for patients to actively participate in nursing care. Although some of the outcomes have high heterogeneity (*I*^*2*^ = 78%–98%), they still maintain statistical significance through random effects model analysis, indicating that the research results are clinically robust. Among them, the low heterogeneity of complication rate (*I*^*2*^ = 34%) and satisfaction (*I*^*2*^ = 0%) suggests that these two indicators have good consistency in different studies (Table 5).

**Table 5:** Summary of key quantitative outcomes from meta-analysis

Outcomes	Effect size	95% CI	*Z*	*P*-value	*I*^2^	Model	Clinical interpretation
Self-management abilities	MD=17.77	13.99–21.55	9.2	<0.00001	98%	Random	Better self-management in the intervention group.
Anxiety	MD=−8.53	−12.19–4.86	4.56	<0.00001	78%	Random	Reduced anxiety in the intervention group.
Depression	MD=−11	−16.43–5.58	3.97	<0.0001	88%	Random	Reduced depression in the intervention group.
Complication incidence	OR=0.17	0.13–0.23	11.79	<0.00001	34%	Fixed	Lower odds of complications in the intervention group.
Compliance	OR=0.16	0.08–0.32	4.98	<0.00001	0%	Fixed	Higher odds of treatment compliance in the intervention group.
Self-efficacy	MD=9.45	1.77–17.13	2.41	0.02	95%	Random	Increased self-efficacy in the intervention group
Satisfaction	OR=6.01	3.40–10.59	6.19	<0.00001	0%	Fixed	Higher odds of satisfaction with care in the intervention group.
Quality of life	MD=9.38	5.19–13.57	4.39	<0.0001	94%	Random	Better quality of life in the intervention group.

*MD* mean difference, *OR* odds ratio. Negative MD values for anxiety/depression indicate improvement

Model selection criteria:

Fixed effects model: *I*^2^ < 50% and *P* > 0.05

Random effects model: *I*^2^ ≥ 50% or *P* ≤ 0.05

*CI* confidence interval, *Z* test statistic, *P-value*, statistical significance

## Discussion

### Home care-based education components

Comprehending the introduction of PICC, the catheterization procedure, and precautions during catheterization is essential for patients and their families to deliver proper home care. Research conducted by Bertoglio S et al. [3] revealed a significant correlation between patients’ understanding of catheter care and the occurrence of catheter-related complications. A study done by Petit et al. [35] designed an educational approach for cancer patients with PICCs by three domains: knowledge, know-how, and attitudes. These findings align with the emphasis on knowledge acquisition highlighted in the catheter care section of this review, underscoring the critical importance of possessing thorough catheter care knowledge to promote the secure and efficient utilization of PICC catheters in patients.

Our review results indicate that providing patients with self-care education is necessary. Initially, instructing individuals on their daily activities can help prevent unhealthy practices that may impact catheter functionality and personal well-being. Additionally, educating patients on emergency preparedness, catheter self-assessment, and complication prevention empowers them to promptly identify and address potential issues, minimize the severity of complications, and enhance the safety of their treatment. A systematic review conducted by Dineen-Griffin S and colleagues [36] revealed that the implementation of successful self-management support techniques plays a crucial role in enhancing patient health results. These results provide strong evidence for the results of our review. A meta-analysis by Bosun-Arije [37] concluded that self-care interventions, including daily activity guidance and complication self-monitoring, achieved physical improvements and reduced the occurrence of psychological problems.

Our review mentioned that patient education also needs to include resource support. Integrating family and hospital resources can build a comprehensive support network for patients. Psychological support, as an important part of resource support, helps alleviate patients’ anxiety, depression, and other negative emotions caused by cancer and long-term tubes, thereby improving their compliance with treatment and care and promoting the overall recovery process. These results can be confirmed by previous studies [9, 38].

Our review’s identification of three core education components aligns with a prior systematic review [39], which conducts patient self-management education with similar components. Additionally, recent studies emphasize the role of digital methods in health education. A scoping review done by Shaffer et al. [40] demonstrated that digital education modules combined with telehealth support significantly improved patients’ knowledge of PICC care and reduced complication rates.

### Meta-analysis results of the home care-based education

#### Discussion on meta-analysis of outcomes

The meta-analysis of the included studies yielded several significant findings that have important implications for the care of cancer patients with PICC lines.

Regarding self-management abilities, the results clearly demonstrate that patients who received home care-based education had a substantial enhancement and improvement. This finding is consistent with the concept that comprehensive education equips patients with the knowledge and skills necessary for effective self-management. By understanding the proper maintenance of the PICC line, self-observation for potential complications, and appropriate lifestyle adjustments, patients are better able to take an active role in their treatment and recovery. This finding is in line with previous research that has emphasized the importance of patient education in promoting self-management and improving health outcomes in chronic disease management [38, 39].

The analysis of mental status also revealed important insights. The significant reduction in anxiety and depression among patients who received home care-based education highlights the psychological benefits. Cancer patients often experience significant psychological distress due to the nature of their disease and the challenges associated with treatment [41]. The home care-based education helped patients better understand and cope with the presence of the PICC line, reducing their anxiety and depression levels. This finding is supported by prior study that has shown the positive impact of patient education and psychological support on mental health in cancer patients [42].

The lower complication incidence in the intervention group indicates that home care-based education plays a significant role in preventing catheter-related complications. By educating patients about the importance of proper catheter care, including regular maintenance, self-observation, and appropriate handling of emergencies, complications such as infection, thrombosis, and catheter detachment can be reduced. This finding is consistent with previous research that has emphasized the importance of patient education in preventing medical device-related complications [43].

The higher compliance rate in patients receiving home care-based education is also of great significance. Compliance with recommended care regimens is essential for the successful management of PICC lines and the overall treatment of cancer patients. The home care-based education increased patients’ understanding of the importance of following the prescribed care plan, leading to improved compliance. This finding is in line with previous research that has shown the positive relationship between patient education and treatment adherence [44, 45].

The improvement in self-efficacy among patients who received home care-based education further emphasizes the positive impact of such programs. Self-efficacy is an important psychological construct that influences patients’ motivation and ability to engage in self-care behaviors [46]. By providing patients with the necessary knowledge and skills, home care-based education can enhance their confidence in managing their health, leading to increased self-efficacy. This finding is consistent with previous research on the role of patient education in promoting self-efficacy in chronic disease management [47].

Finally, the higher patient satisfaction with nursing care services in the intervention group indicates that comprehensive home care-based education not only improves clinical outcomes but also enhances the patient experience. This is an important aspect of healthcare quality, as satisfied patients are more likely to adhere to treatment, have better communication with healthcare providers, and experience improved overall well-being. This finding is in line with previous research that has emphasized the importance of patient satisfaction in healthcare delivery [48].

#### Discussion on the source of heterogeneity

This study found high heterogeneity in self-management ability (*I*^2^ = 98%) and quality of life (*I*^2^ = 94%), which may be related to the following factors:

- Different education protocols: The included studies adopted a variety of education protocols, including face-to-face education [22, 24, 27, 33], face-to-face + telephone follow-up [11, 17, 19, 30], WeChat platform education [21, 23, 25, 26, 31, 32], face-to-face + WeChat [18, 29], WeChat + telephone follow-up [28], and home visits [20]. Among them, WeChat-based education programs improved the efficiency of information transmission through digital education materials, while traditional face-to-face education may have different effects due to geographical and time restrictions. This diversity of intervention forms may explain the significant heterogeneity of self-management ability.
- Different follow-up time: The follow-up period of the studies ranged from 1 to 6 months. Short-term follow-up may make it easier to observe behavioral changes in the early stages of education, while long-term follow-up may reflect the attenuation of the effect of continuous intervention [49]. For example, a 19.3-point improvement in self-management ability showed after a 3-month-intervention [22], while only a 2.62-point improvement showed after a 6-month-intervention [25]. This time effect may exacerbate heterogeneity.
- Differences in participant characteristics: Among the included studies, 89.5% of the samples were from China, and there was only one Australian study (Sharp et al., 2024). The age of patients in Chinese studies was concentrated between 45 and 60, while the average age of Australian samples was 62. Differences in cultural background and digital literacy may lead to different educational effects.
- Differences in intervention contents: Educational content ranges from basic nursing knowledge to psychological support. For example, studies that included psychological interventions [24] showed more significant improvements in depression (MD = −18.26), while studies that only provided technical guidance [28] showed weaker effects (MD = −8.79). This difference in intervention depth may lead to heterogeneity.

#### Handling of missing data

Of the 19 studies included in this study, only five (26%) clearly reported the treatment methods for missing data: Three RCT studies [20, 27, 33] used intention-to-treat (ITT) analysis and included participants who dropped out after randomization in the original group for analysis. Two quasi-experimental studies [21, 32] used per-protocol (PP) analysis and only included participants who completed all interventions. According to RoB 2 tool assessment results, 86% of RCTs were at low risk for incomplete outcome data, for the dropout rate in most studies was ≤10%. However, there are still 2 studies [22, 28] that did not report the dropout rate, which may affect the accuracy of the results.

### Limitations

While this study reveals the positive effects of home care-based education on cancer patients with PICCs, it also has limitations that need to be paid attention to. Of the 19 included studies, 18 were from China and only one was from Australia. This regional concentration makes the generalizability of the research results questionable. There are significant differences between different countries in the operation mode of the medical system, the implementation methods of patient education, and the social and cultural background: China’s unique family-involved nursing model, remote guidance based on WeChat social media platform, and the allocation of primary medical resources are fundamentally different from the actual situation in other countries. Although studies have confirmed that home care-based education has several positive effects, the intervention effect may be greatly reduced when the medical support system, patient family structure, or technology application scenario on which the education strategy relies changes.

The inclusion criteria restricted studies to those published in English or Chinese, introducing potential language bias. Although comprehensive searches were conducted in major databases, excluding studies published in other languages may have missed relevant evidence from non-Asian regions. Studies from other regions might yield different results due to variations in healthcare systems, religions, and culture.

## Conclusion

For patients with PICC, healthcare providers can provide home care-based education on catheter care, self-care, and resource support. By conducting home care-based education, patients’ self-management ability, mental health, complications, compliance, self-efficacy, satisfaction, and quality of life have all been significantly improved.

While this review employed an extensive search strategy, utilizing various Chinese and English databases, and performed a rigorous quality assessment of the included literature to guarantee the accuracy and dependability of the findings, it is important to acknowledge that this study does have certain constraints. The majority of the included studies were from China, which may limit the generalizability of the results to other regions and cultures. Second, the exclusive inclusion of English and Chinese studies introduces language bias, as evidence from non-Asian regions may differ due to healthcare system variations. Third, high heterogeneity in outcomes like self-management (*I*^2^ = 98%) suggests intervention protocols require standardization.

In terms of future research, it is recommended to focus on validating the effectiveness of the developed education list in different countries and cultural groups. It is also recommended that subsequent studies focus on cross-cultural comparative analysis, verify the applicability of the education program at different levels of development such as in Europe, America, and Africa, and focus on building a culturally sensitive education content framework, so as to enhance the international promotion value of the intervention strategy. Additionally, future systematic reviews are recommended to consider including multiple languages or collaborate with translators to mitigate language bias. In clinical practice, the education components can serve as a valuable resource for nurses and other healthcare providers. Prioritizing methodologically rigorous designs should be considered to develop standardized education protocols incorporating digital tools to enhance accessibility and generalizability.

## Data Availability

No datasets were generated or analysed during the current study.
